# A POLICY COMPARISON OF THE EVOLUTION OF COMMUNITY-BASED LONG-TERM CARE POLICY BETWEEN TAIWAN AND THE UNITED STATES

**DOI:** 10.1093/geroni/igad104.2891

**Published:** 2023-12-21

**Authors:** Allie Peckham, Li-Chuan Liu, Laura Schmid

**Affiliations:** Arizona State University, Phoenix, Arizona, United States; National Taitung University, Taitung, Taitung, Taiwan (Republic of China); Arizona State University, Phoenix, Arizona, United States

## Abstract

Governments globally have been facing pressure to develop and implement effective policies to support population aging. This approach was historically rooted in a perception that as we age we become increasingly incapacitated. This research seeks to understand how policies in two countries valuing unpaid care differently, developed and implemented Long-term services and supports (LTSS). We compare the federal level efforts of implementing LTSS within the United States (U.S.) and Taiwan. Relying on a pre-established comparative framework developed by Richard Rose, we utilized a deductive analytical approach to assess the differences in policy options and timelines across Taiwan and the U.S.. After attending this session, participants will develop a deeper understanding of how Taiwan and the U.S. are working to offer home and community-based long-term care services. It wasn’t until the 1980s for the U.S. and late 1990s for Taiwan that LTSS were introduced with the intention of supporting people to live as independently as possible in their own homes for as long as possible (e.g., Department of Health and Human Services, 3-year Care Service Development Plan). It is likely that the delay in LTSS delivery between the two countries is rooted in the role that unpaid support is expected to play. There is increasing evidence that countries are shifting their policy efforts to focus less on aging as a social dependency and more on policies that encourage healthy longevity.

